# Targeting P2Y_12_ limits T_H_17 accumulation in the CNS and reduces EAE progression

**DOI:** 10.3389/fimmu.2026.1946414

**Published:** 2026-10-21

**Authors:** Niklas Huntemann, Lara-Maria Preuth, Thorsten F. Krug, Anna Vogelsang, Linda Groeneweg, Paul Disse, Rajeevan Narayanan Therpurakal, Alexander Zarbock, Bernhard Nieswandt, Kerstin Göbel, Harald F. Langer, Tobias Ruck, Antje Schmidt-Pogoda, Amin Polzin, Susann Eichler, Sven G. Meuth

**Affiliations:** 1 Department of Neurology, University Hospital Münster, Münster, Germany; 2 Department of Neurology, University Hospital Düsseldorf, Heinrich Heine University Düsseldorf, Düsseldorf, Germany; 3 Department of Radiology, Clemenshospital GmbH Münster, Münster, Germany; 4 Institute of Immunology, University of Münster, Münster, Germany; 5 Department of Anaesthesiology, Intensive Care and Pain Medicine, University of Münster, Münster, Germany; 6 Rudolf Virchow Center, Deutsche Forschungsgemeinschaft Research Center for Experimental Biomedicine, University of Würzburg, Würzburg, Germany; 7 Department of Medicine, Division of Cardiology, Angiology, Hemostasis, and Medical Intensive Care, Medical Faculty Mannheim, University of Heidelberg, Mannheim, Germany; 8 Department of Neurology, BG University Hospital Bergmannsheil, Ruhr University Bochum, Bochum, Germany; 9 BG University Hospital Bergmannsheil, Heimer Institute for Muscle Research, Bochum, Germany; 10 Division of Cardiology, Pulmonology, and Vascular Medicine, Heinrich Heine University Medical Center Düsseldorf, Düsseldorf, Germany; 11 Cardiovascular Research Institute Düsseldorf (CARID), Düsseldorf, Germany; 12 Institute for Laboratory Medicine, Microbiology and Clinical Pathobiochemistry, Bielefeld University, Medical School and University Medical Center OWL - Campus Klinikum Lippe, Detmold, Germany; 13 Department of Science and Academic Medicine, Bielefeld University, Medical School and University Medical Center OWL - Campus Klinikum Lippe, Detmold, Germany

**Keywords:** clopidogrel, EAE, elinogrel, MS, P2Y12, platelets, TH17

## Abstract

Platelets are increasingly recognized as active amplifiers of neuroinflammation in multiple sclerosis and experimental autoimmune encephalomyelitis (EAE). P2Y12 is an essential ADP receptor that regulates platelet activation and is upregulated in neuroinflammatory lesions. However, its contribution across different effector populations and therapeutic potential in autoimmune diseases of the central nervous system (CNS) remain incompletely defined. Therefore, we characterized P2ry12 expression across different immune and CNS-resident effector populations involved in EAE. To define P2Y12-dependent mechanisms in autoimmune neuroinflammation, we targeted the P2Y12 receptor both genetically and pharmacologically in MOG35–55-induced EAE and monitored disease course and immunopathology. The neuroinflammatory response was profiled by flow cytometry, histology, and cytokine analyses. Pharmacological inhibition with elinogrel or clopidogrel was evaluated in prophylactic and therapeutic treatment regimens. Additionally, prophylactic elinogrel treatment was combined with antibody-mediated platelet depletion during the effector phase of EAE to define the contribution of platelets to the observed effects. Among the immune and CNS-resident effector populations examined, platelets exhibited the highest abundance of P2ry12 transcripts, and platelet P2ry12 expression increased following EAE induction. Consistent with a proinflammatory role of the P2Y12 receptor in CNS autoimmunity, its genetic deficiency was associated with decreased disease progression, as indicated by lower peak and cumulative scores, while disease onset remained unchanged compared to wild-type mice. Pharmacological inhibition of the P2Y12 receptor reproduced this protective effect. Importantly, treatment with elinogrel did not affect the number of infiltrated platelets, but markedly reduced cellular infiltration and demyelination. Furthermore, the protective effect was associated with a significant reduction in infiltrating CD4+ T cells, particularly pro-inflammatory T helper 17 (TH17) cells. Of note, elinogrel provided no additional benefit beyond platelet depletion, a finding compatible with a platelet-mediated contribution to the observed effect. Clopidogrel, another P2Y12 inhibitor, similarly reduced disease severity when administered prophylactically. Of note, both elinogrel and clopidogrel remained effective when treatment was initiated after clinical onset. These data identify platelet-associated P2Y12 signaling as a functionally relevant amplifier of EAE pathology. P2Y12 blockade limits CNS immune infiltration and neuroinflammation-induced tissue injury, exerts an impact on the TH17 axis, and retains efficacy when administered therapeutically. Collectively, these findings support P2Y12 as a mechanistically grounded and translationally relevant target in CNS autoimmunity.

## 1 Introduction

Multiple sclerosis (MS) is a chronic inflammatory disease of the central nervous system (CNS) characterized by immune cell infiltration, demyelination, and neuroaxonal damage (1, 2). An early pathogenic hallmark of MS pathophysiology is lymphocyte transmigration across a compromised blood–brain barrier (BBB), which initiates and sustains neuroinflammation throughout disease progression (3, 4). Experimental autoimmune encephalomyelitis (EAE) is a widely recognized preclinical model of MS that reflects central immunopathological characteristics of the human condition (5–7). This model has yielded significant mechanistic insights into CNS-directed autoimmunity.

Beyond the classical components of the immune system, accumulating evidence indicates that additional cellular players contribute to MS immunopathophysiology (8–15). Among these, platelets have emerged as important immunomodulators. While historically regarded primarily as mediators of haemostasis, platelets have increasingly been recognized as active regulators of inflammatory and (auto-)immune responses (16–18). Despite lacking a nucleus, platelets possess a highly organized cytoskeleton, functional mitochondria, an extensive membrane system, and both α-granules and dense granules which contain different pro-inflammatory mediators (19). In addition, they harbor diverse RNA species, including a large reservoir of mRNA, as well as components of the spliceosome machinery (19). Together, these features enable rapid and stimulus-dependent platelet responses to inflammatory cues (8). Functionally, once activated, platelets influence endothelial and immune cells by engaging in direct cellular interactions and by releasing bioactive substances from α-granules and dense granules (10, 17). Although platelet involvement has been demonstrated in several autoimmune diseases (8), their contribution to autoimmune neuroinflammation has remained less clearly defined. Nonetheless, compelling evidence suggests that platelets participate in shaping aberrant CNS-directed adaptive immune responses, particularly at early stages of disease (8–13). Beyond their effects on peripheral immune responses, platelets have been shown to infiltrate the CNS and accumulate within lesions early in the course of EAE (9–13), a process that is also evident in human MS lesions (9). Recent findings in EAE suggest that platelets accumulate in the CNS at very early stages of neuroinflammation and may precede T-cell entry in certain compartments. This observation supports the concept that platelets are rapidly recruited to sites of neurovascular injury, potentially as part of their haemostatic function, before substantial lymphocytic infiltration occurs (11, 12). Also in MS, circulating platelets exhibit an increased activation state (18, 20, 21). Early depletion of platelets has been shown to reduce disease severity and decrease recruitment of leukocytes to the CNS (9, 10, 12, 13). Together, these findings support a substantial contribution of platelet activity to neuroinflammatory lesion development.

Adenosine diphosphate (ADP) signaling via the purinergic P2Y_12_ receptor represents a plausible link between platelet activation and immune modulation in neuroinflammation (22–24). P2Y_12_ serves as a key amplifier of platelet activation, with its ligand ADP being released passively from damaged endothelium and actively from dense granules of platelets that have been activated (25–27). This dual release mechanism potentially reinforces thrombo-inflammatory signaling locally at sites of neurovascular damage. Elevated P2Y_12_ expression has been observed in models of neuroinflammation (22, 24), while pharmacological inhibition of P2Y_12_ receptors using clopidogrel and ticagrelor has demonstrated beneficial effects in EAE (24). Regarding genetic deficiency of P2Y_12_, the available data are heterogeneous. On the one hand, it has been shown that genetic depletion of P2Y_12_ leads to a milder disease course, mediated by reduced activation of the T helper 17 (T_H_17) cell/interleukin (IL)-17A axis, along with attenuated CNS infiltration and demyelination (24). In contrast, another study reported exacerbated EAE progression following global genetic P2Y_12_ deficiency, which was attributed to increased IL-23 production by dendritic cells (28). Moreover, the specific cellular compartment responsible for these effects and the relevance of P2Y12-targeted therapeutic interventions initiated after disease onset remain unclear.

This study investigated the contribution of platelet-associated P2Y_12_ signaling to EAE pathogenesis using complementary genetic and pharmacological approaches. Studies were performed in *P2ry12*-deficient mice and complemented by pharmacological inhibition and platelet depletion models to determine the platelet dependence of the observed phenotype. Downstream immunological correlates of prophylactic P2Y_12_ inhibition were assessed using flow cytometry, immunohistochemistry, and cytokine analyses. In addition, we evaluated the therapeutic efficacy of pharmacological P2Y_12_ inhibition initiated at the onset of clinical disease. This framework enabled analysis of the underlying mechanism and the translational potential of targeting the P2Y_12_ axis in CNS autoimmunity.

## 2 Materials and methods

### 2.1 Study approval

All animal experiments received approval from the regulatory authorities for animal welfare and scientific research in North Rhine-Westphalia, Germany (81-02.04.2018.A382, 84-02.04.2013.A142, 84-02.05.50.17.019). Experiments were conducted in full compliance with applicable laws and regulations as well as the ARRIVE guidelines (Animal Research: Reporting of *In Vivo* Experiments).

### 2.2 Mice

C57BL/6J mice were obtained from Charles River Laboratories (Sulzfeld, Germany). P2Y_12-_deficient (*P2ry12^-/-^*) mice were purchased from Deltagen Inc. (USA). All strains were bred on a C57BL/6J background, and genotypes were confirmed by polymerase chain reaction (not shown). All animals were maintained under standardized conditions, with access to water and food *ad libitum*.

### 2.3 EAE induction and treatment

To induce active EAE, female mice aged 8–12 weeks were immunised with 200 μg of murine myelin oligodendrocyte glycoprotein (MOG)_35–55_ peptide (Sequence: MEVGWYRSPFSRVVHLYRNGK; purity ≥ 99%, verified by high-performance liquid chromatography; sourced from Charité – Universitätsmedizin Berlin, Berlin, Germany). This peptide was mixed with 200 µl of complete Freund’s adjuvant (Merck, Darmstadt, Germany), which contained 200 µg of Mycobacterium tuberculosis (strain H37Ra; Becton, Dickinson and Company [BD], Sparks, USA). MOG_35–55_ emulsion was administered subcutaneously at two sites near the inguinal lymph nodes, with a volume of 100 µl per site, under isoflurane anesthesia. On days 0 and 2 after immunization, 100 ng of pertussis toxin (lot number 1001, potency 0.6; Hooke Laboratories, Lawrence, MA, USA) was injected intraperitoneally (*i.p.*) in 100 μl of Dulbecco’s phosphate-buffered saline (DPBS; Merck), which did not contain CaCl_2_ or MgCl_2_. Age-matched mice were used for all experiments. When therapeutics were administered to mice of the same genotype, animals were randomly assigned to the respective experimental groups. Treatment group animals were either treated with a 40 mg/kg body weight dose of elinogrel (Novartis, Basel, Switzerland) or clopidogrel (Merck) *via* oral gavage. Elinogrel was dissolved in 4% dimethyl sulfoxide (DMSO)/0.5% aqueous carboxymethyl cellulose (CMC) and clopidogrel in DPBS/0.5% aqueous CMC. As vehicle controls, DMSO/CMC or DPBS/CMC aqueous solutions without elinogrel or clopidogrel, respectively, were administered orally to the respective control groups (500 µl per animal). Platelet depletion was performed as previously described by our group (9). In detail, mice were injected *i.p.* with anti-platelet serum (rabbit anti-mouse thrombocyte; Wak-Chemie, Steinbach/Taunus, Germany) or control serum (normal rabbit serum; Wak-Chemie) at 0.5 ml/kg body weight in 100 µl DPBS on days 12 and 16 after EAE induction.

### 2.4 Evaluation of EAE

Clinical EAE signs were assessed daily by two independent observers blinded to treatment allocation and mouse genotype, as applicable. These signs were assigned clinical scores as follows: grade 0 indicated no observable symptoms; grade 1, partially limp tail; grade 2, fully paralyzed tail; grade 3, moderate weakness in the hindlimbs; grade 4, complete hindlimb weakness; grade 5, mild paraparesis; grade 6, paraparesis with forelimb weakness; grade 7, severe paraparesis or paraplegia; grade 8, tetraparesis; grade 9, quadriplegia or a pre-moribund state; and grade 10, death. Mice were considered to have developed EAE and were therefore included in the analysis if they had a score of at least 1 for more than one day in a row. Animals with a score worse than 7 or with weight loss exceeding 20% of the initial body weight were taken out of the experiment to guarantee animal welfare, and their last observed score was assigned for the remainder of the experiment. Cumulative disease scores were defined as the mean daily clinical score per mouse over the observation period. Weight loss was measured as the change from initial weight post-immunization and shown as relative body weight over time.

### 2.5 Histopathology and immunohistochemistry

Following transcardial perfusion, spinal cords were excised, embedded in a cryoprotective medium (Tissue-Tek O.C.T.; Science Services, Munich, Germany), and subsequently sectioned using a cryotome (Leica, Wetzlar, Germany). Sections measuring 10 µm in thickness were stained with hematoxylin and eosin (H&E) for the evaluation of inflammation, or with FluoroMyelin Green (1:300; Thermo Fisher, Waltham, USA) to selectively label myelin and assess the extent of demyelination, in accordance with established protocols. To identify platelets or Ionized calcium-binding adapter molecule 1 (Iba1)-positive cells, tissue sections were post-fixed with 4% paraformaldehyde (PFA) for 10 minutes at room temperature (RT), followed by three washing steps with DPBS. Samples were then blocked for one hour at RT using DPBS supplemented with 5% bovine serum albumin (BSA) and 1% normal goat serum. Sections were then incubated overnight at 4 °C with primary antibodies: rat anti-mouse Cluster of differentiation (CD)42c (1:100; clone 3G6; provided by B. Nieswandt, Würzburg, Germany) and rabbit anti-mouse CD31 (1:100; polyclonal; Abcam, Cambridge, GB) for platelet staining, or rabbit anti-mouse Iba1 (1:250; polyclonal; Wako Chemicals GmbH, Neuss, Germany) for macrophage/microglia staining. Each antibody was diluted in DPBS containing 5% BSA and 1% normal goat serum. Following washing steps, sections were incubated with secondary antibodies Cy2-coupled goat anti-rat and Cy3-coupled goat anti-rabbit (1:800; polyclonal, Dianova, Hamburg, Germany) diluted in DPBS for 1 hour at RT. Negative controls were obtained by either omitting the primary or secondary antibody and revealed no detectable signal (not shown). For visualizing cell nuclei within immunofluorescent staining, sections were embedded in Fluoromount-G with 4’, 6-diamidino-2-phenylindole (DAPI; Thermo Fisher) prior to microscopic analysis. Stained sections were examined by microscopy (Axiophot2, Zeiss, Oberkochen, Germany) with a charge-coupled device camera (Zeiss) and analyzed in a blinded manner using Axiovision (Zeiss) and ImageJ software (National Institute of Mental Health, Bethesda, USA). Finally, inflammatory as well as demyelinated areas and CD42c^+^ as well as Iba1^+^ cells were quantified on 5 randomly selected coverslips, each with three sections of 6 to 9 mice per group, and expressed as the percentage of total area or number of positive cells/mm^2^. For each animal, the arithmetic mean was determined from the related sections.

### 2.6 Cell preparation and isolation

CNS-infiltrating cells and platelets were purified from brains and spinal cords collected after thorough transcardial perfusion with DPBS. CNS tissue was cut into pieces and further mechanically homogenized in DPBS, layered on a 30-50% Percoll (Merck) gradient, and centrifuged for 30 min at 1,200 g without brake. To accurately determine the number of cells isolated at the interphase, counting beads (BD) were added to freshly purified cells before proceeding with flow cytometry staining. For platelets, CNS tissue was centrifuged at low speed (250 x g) for 5 min after homogenization to obtain platelet-rich supernatant as shown (10).

Cell populations analyzed for *P2ry12* mRNA expression were obtained from naïve Wild-type (WT) C57BL/6J mice. Peripheral platelets were isolated from sodium citrate anticoagulated blood collected from the retro orbital plexus. Platelet rich plasma was obtained by centrifugation at 300 × g for 10 min at RT. Following a second extraction with HEPES Tyrode buffer, the combined platelet rich fractions were centrifuged at 1000 × g for 5 min. Platelets were washed and lysed in TRIzol Reagent (Thermo Fisher Scientific) for RNA extraction. Platelet identity was confirmed by flow cytometry as CD41^+^CD61^+^ events (antibodies purchased from BioLegend).

For immune cell isolation, spleens were mechanically dissociated and passed twice through 40 µm cell strainers, followed by erythrocyte lysis and washing. CD4^+^ T cells, CD8^+^ T cells, B cells, CD11c^+^ dendritic cells and macrophages were purified from the resulting splenic single cell suspensions by magnetic activated cell sorting using the respective mouse isolation kits according to the manufacturer’s instructions (Miltenyi Biotec, Bergisch Gladbach, Germany).

Murine brain microvascular endothelial cells were prepared from the cortices of naïve adult C57BL/6J mice. Following removal of the meninges, cortical tissue was digested with collagenase type II (Worthington) and DNase I (Sigma Aldrich). Myelin was removed by density separation in BSA, followed by a second digestion with collagenase and dispase (Roche) and enrichment of the microvascular fraction on a Percoll gradient (Merck). Cells were cultured for five days on collagen IV and fibronectin-coated surfaces (Sigma Aldrich), with short-term puromycin selection to reduce non-endothelial contaminants.

Primary microglia were generated from mixed glial cultures prepared from the brains of postnatal day 1 to 5 mice. Cells were maintained in L-glutamine containing DMEM supplemented with 10% heat inactivated FCS, 1% non-essential amino acids and 1% penicillin and streptomycin in poly-L-lysine-coated flasks (Sigma Aldrich). After 14 days, microglia were selectively detached by gently tapping the culture flasks.

Primary astrocytes were prepared from the cortices of postnatal day 1 to 3 mice. Cortical tissue was incubated with DNase I and 2.5% trypsin for 15 min at 37 °C, mechanically dissociated and filtered through 45 µm cell strainers. Cells were cultured in MEM supplemented with sodium pyruvate, glucose, horse serum and penicillin and streptomycin. Confluent astrocytes were subsequently maintained on poly-D-lysine-coated surfaces.

Oligodendrocyte precursor cells (OPC) were isolated from the cerebra of postnatal day 6 to 9 mice by sequential immunopanning. Negative selection was performed on plates coated with BSL1 Griffonia simplicifolia lectin (Vector Laboratories), followed by positive selection on anti-CD140a-coated plates (BioLegend). Bound cells were detached by scraping and cultured on poly-L-lysine-coated surfaces (Sigma Aldrich) in Sato medium supplemented with PDGF AA and neurotrophin 3 (PeproTech). Mature oligodendrocytes were generated by replacing PDGF AA with CNTF (PeproTech) for two days.

Primary hippocampal neurons were prepared from embryonic day 18 C57BL/6 mouse embryos. Hippocampi were incubated in 0.25% trypsin in HBSS (Invitrogen) for 5 min at 37 °C, washed and mechanically dissociated in neuronal medium containing Modified Eagle’s medium, sodium bicarbonate, sodium pyruvate, L-glutamine and B27 supplement (Invitrogen), glucose (Merck) and penicillin and streptomycin (Biochrom). Cells were seeded on poly-D-lysine-coated surfaces (Sigma Aldrich) and cultured for 5 to 7 days at 37 °C and 5% CO_2_.

Cell identity and purity were routinely assessed by flow cytometry or cell-type-specific immunostaining using CD3, CD4 and CD8 for T cells, B220 for B cells, CD11c for dendritic cells, CD11b and F4/80 for macrophages, CD11b for microglia, GFAP for astrocytes, NeuN for neurons, PDGFRα for OPC and O4 or MBP for mature oligodendrocytes. All preparations included in the analysis had a purity > 90%.

### 2.7 Flow cytometry

To detect CNS-infiltrating platelets, platelet-rich supernatants derived from CNS homogenates were stained using anti-CD41 (MWReg30, BioLegend) and anti-CD61 antibodies (2C9.G2, BioLegend), followed by fixation with 1% PFA prior to analysis. Cells infiltrating the CNS were labelled using suitable combinations of fluorochrome-conjugated antibodies from BioLegend, targeting the following markers: CD3 (17A2), CD4 (GK1.5), CD8a (53-6.7), CD11b (M1/70), CD45 (30-F11), and CD45R/B220 (RA3-6B2). To block Fc receptors, cells were preincubated with purified anti-CD16/CD32 antibodies (BioLegend) following the manufacturer’s recommended protocol. To exclude dead cells, a Zombie fixable viability dye (diluted 1:1000; BioLegend) was applied. For intranuclear Forkhead box protein P3 (FoxP3) analyses (FJK-16S, Thermo Fisher), cells were fixed/permeabilized with the FoxP3/Transcription Factor Staining Buffer Set (Thermo Fisher). For cytokine analyses, cells were polyclonally restimulated for 4 h at 37 °C in the presence of phorbol 12-myristate 13-acetate/ionomycin as well as brefeldin A using a cell activation cocktail (BioLegend) to block cytokine secretion. After fixation and permeabilization with Cyto-Fast Fix/Perm Buffer Set (BioLegend), intracellular staining was performed using antibodies against interferon-γ (IFN-γ; clone XMG1.2, BioLegend) and IL-17A (clone eBio17B7, Thermo Fisher Scientific). Stained samples were analyzed using a multi-color flow cytometer (Gallios, Beckman Coulter, Krefeld, Germany).

### 2.8 *Ex vivo* platelet activation assay

Platelets were incubated with 0, 1, 10, or 100 µM elinogrel and were left unstimulated or stimulated with ADP. Surface expression of CD62P and integrin αIIb was determined using antibodies from Emfret Analytics (Eibelstadt, Germany). Staining was performed for 15 min at RT in the dark. Samples were analyzed on a Gallios flow cytometer (Beckman Coulter, Krefeld, Germany).

### 2.9 Quantitative real-time polymerase chain reaction

Total RNA was extracted using TRIzol (Thermo Fisher), then converted to cDNA with Maxima reverse transcriptase. The resulting cDNA served as a template for RT-PCR, which was carried out with Maxima probe/ROX qPCR mastermix, following the manufacturer’s guidelines on the Applied Biosystems StepOne Plus RT-PCR system (Thermo Fisher). *P2ry12* expression was measured using the TaqMan Gene Expression Assay *Mm00446026_m1* and normalized to the housekeeping gene (18S rRNA). Results were analyzed using the StepOne software (Thermo Fisher) and the comparative Ct method (Ct is defined as the cycle threshold). Data are presented as ΔCt values, where ΔCt is defined by subtracting the Ct of the housekeeping gene from the Ct of the target gene. All data points were measured in triplicate, and mean Ct values were used for calculations.

### 2.10 Proliferation assay and cytokine assays

To assess MOG_35-55_-induced immune cell proliferation, adenosine triphosphate (ATP) was measured using the ATPlite™ luminescence assay (PerkinElmer) according to the manufacturer’s instructions. Cells were lysed with 50 µL mammalian cell lysis solution for 5 min, followed by addition of 50 µL substrate solution containing luciferin/luciferase and incubation for 5 min. Plates were incubated in the dark for 10 min before luminescence was read on a photometer (Infinite^®^ M200Pro). Triplicates were averaged and the proliferation index was calculated as the ratio of stimulated to unstimulated mean luminescence.

For cytokine analysis, 3 × 10^6^ splenocytes in 1 mL were plated in duplicate and cultured with 10 µg/mL MOG_35–55_ or medium alone. After 48 h, cell-free supernatants were collected following centrifugation for 10 min at 4 °C and stored at −20 °C until analysis. Concentrations of IL-6 (detection limit: 4 pg/mL), IL-10 (detection limit: 32 pg/mL), IL-17A (detection limit: 4 pg/mL), IFN-γ (detection limit: 15 pg/mL), TNF-α (detection limit: 8 pg/mL), TGF-β1 (detection limit: 8 pg/mL) and IL-23 (detection limit: 15 pg/mL) were determined using mouse ELISA assays from eBioscience according to the manufacturer’s instructions. Samples analyzed for TGF-β1 were acid-activated before measurement. Absorbance was measured at 450 nm and cytokine concentrations were calculated from eight-point standard curves using four-parameter logistic regression after subtraction of blank values. Duplicate measurements were averaged. Each mouse represented one biological replicate and data were obtained from two independent experiments.

### 2.11 Statistical analysis

Details regarding group sizes and statistical analyses for each experiment are provided in the corresponding figure legends. Unless otherwise specified, data are expressed as the mean ± standard error of the mean from a minimum of three independent experiments. Data were analyzed using Prism 5.04 (GraphPad Software, San Diego, USA), and values of probability (*P*) < 0.05 were considered statistically significant. The level of significance was indicated as NS (not significant), * *P* < 0.05, ** *P* < 0.01, or *** *P* < 0.001.

## 3 Results

### 3.1 P2Y_12_-deficient mice are less prone to an early inflammatory response in EAE

The secretion of platelet granule contents, a process called degranulation, can be triggered by multiple activators and accompanies platelet activation at sites of neurovascular damage (25). In EAE, endothelial injury and platelet activation are associated with local release of ADP, a key ligand for the P2Y_12_ receptor (27). Given conflicting previous reports on the role of P2Y_12_ in EAE (24, 28), *P2ry12* expression across cell types implicated in EAE was profiled first. Among these cell populations, platelets showed the highest *P2ry12* expression compared with peripheral immune and CNS-resident cell types (**Figure 1A**). Following EAE induction (**Figure 1B**), platelets from immunized WT mice displayed increased *P2ry12* transcript levels compared with platelets from naïve controls (naïve ΔCt 10.26 ± 0.28 and EAE 8.64 ± 0.29; *P =* 0.0087; **Figure 1C**).

**Figure 1:**
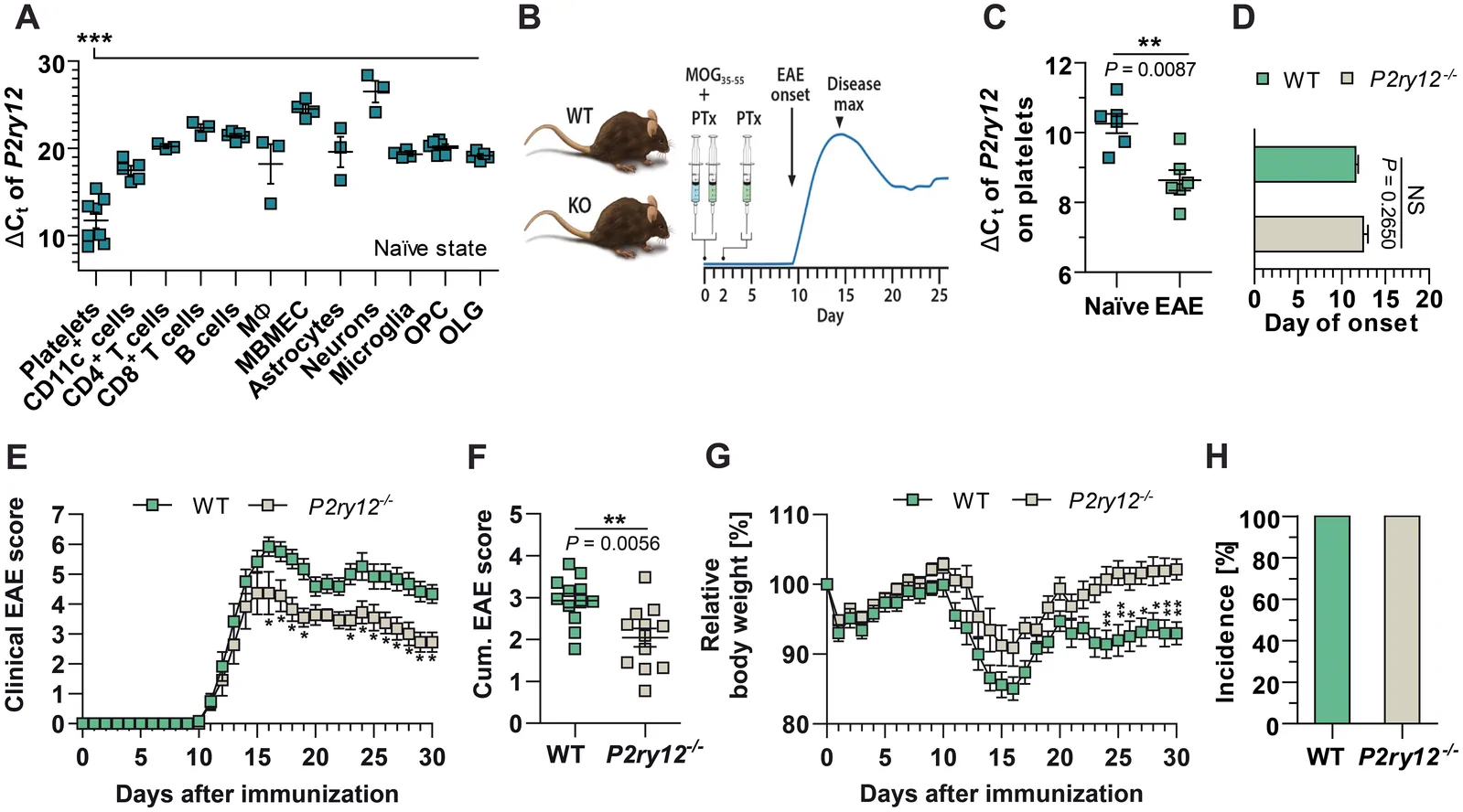
Mice lacking ADP receptor P2Y_12_ are less prone to neuroinflammation. **(A)** Real-time reverse-transcription polymerase chain reaction (rRT-PCR) analyses for *P2ry12* gene expression are shown as ΔCt in naïve platelets and various naïve immune or CNS-resident cell subsets. **(B)** Experimental design of active experimental autoimmune encephalomyelitis (EAE) induction in wild-type (WT) and knockout (KO) mice illustrating the time points of myelin oligodendrocyte glycoprotein (MOG)_35–55_ peptide and pertussis toxin (PTx) administration. **(C)** *P2ry12* gene expression was measured in both naïve platelets and those isolated from wild-type mice on day 16 after active EAE induction (n = 6 per experimental group). The results are reported as ΔCt values. **(D)** Displayed is the average day of disease onset defined as the first day with a score ≥ 1 after EAE induction in WT and ADP receptor P2Y_12_-deficient (*P2ry12^-/-^*) mice. **(E)** The clinical as well as the **(F)** cumulative (Cum.) EAE scores, **(G)** weight changes over time and **(H)** the percentage of animals that developed EAE symptoms after immunization are shown for both groups (n = 12 mice per group). All data were assembled from two independent experiments. Data were analyzed using a Kruskal-Wallis test followed by Dunn’s multiple-comparisons test for pairwise comparisons between groups **(A)**, by a two-way ANOVA with genotype (WT vs. P2ry12⁻/⁻) and time as factors, followed by Bonferroni’s multiple-comparisons test to compare WT and P2ry12⁻/⁻ mice at each individual time point **(E, G)** or a two-tailed Mann-Whitney *U*-test **(C, D, F)**. Each symbol represents an individual sample **(A)** or mouse **(C, F)**. In **(A)**, each symbol represents one independently generated biological cell preparation (platelets, n = 8; CD11c^+^ cells, n = 5; CD4^+^ T cells, n = 4; CD8^+^ T cells, n = 4; B cells, n = 5; macrophages, n = 3; MBMECs, n = 4; astrocytes, n = 3; neurons, n = 3; microglia, n = 4; OPCs, n = 4; and OLGs, n = 4). Each biological sample was analyzed in technical triplicate, and technical replicates were averaged before statistical analysis. The level of significance was labeled as NS, *P < 0.05, **P < 0.01, or *** *P* < 0.001. ADP, adenosine diphosphate; ANOVA, analysis of variance; CNS, central nervous system; Ctrl, control; Cum, cumulative; EAE, experimental autoimmune encephalomyelitis; KO, knockout; MΦ, macrophages; MBMEC, murine brain microvascular endothelial cells; MOG, myelin oligodendrocyte glycoprotein; NS, not significant; OPC, oligodendroglial precursor cells; OLG, oligodendrocytes; PTx, pertussis toxin; rRT-PCR, real-time reverse-transcription polymerase chain reaction; WT, wild-type.

Next, the influence of P2Y_12_ on EAE progression was assessed using mice genetically deficient for the P2Y_12_ receptor. These *P2ry12^-/-^* mice developed clinical signs with a similar onset to that of WT controls (WT 11.58 ± 0.31 and *P2ry12^-/-^* 12.45 ± 0.56 days after immunization; *P =* 0.27; **Figure 1D**). However, consistent with previous results (24), *P2ry12^-/-^* mice reached a significantly lower peak disease severity (**Figure 1E**) and exhibited a reduced cumulative disease score (WT 2.93 ± 0.17 and *P2ry12^-/-^* 2.05 ± 0.22; *P =* 0.0056; **Figure 1F**). In line with this attenuated disease course, *P2ry12^-/-^* mice recovered body weight earlier during the later phase of the disease (**Figure 1G**). All mice of both genotypes developed clinical EAE symptoms (**Figure 1H**).

### 3.2 P2Y_12_ blockade using elinogrel improves EAE course and limits the neuroinflammatory response

Building on these genetic findings, we evaluated whether pharmacological P2Y_12_ inhibition using elinogrel reproduces these effects in EAE. To investigate the pharmacological inhibition of P2Y_12_ signaling, we used elinogrel, a direct, reversible P2Y_12_ inhibitor that does not require metabolic activation (29–31). Compared with prodrug-type inhibitors such as clopidogrel, this profile reduces metabolism-dependent confounding and is therefore suitable for mechanistic testing of the P2Y_12_ pathway in EAE (29). To confirm the pharmacodynamic activity of elinogrel in inhibiting ADP-dependent platelet activation, platelet responses were analyzed *ex vivo*. In murine platelets, elinogrel treatment reduced ADP-induced surface expression of CD62P and integrin αIIb in a dose-dependent manner (**Supplementary Figure 1**). Prophylactic treatment with elinogrel once daily, starting on the day of immunization (**Figure 2A**), significantly attenuated clinical EAE scores (**Figure 2B**) and cumulative scores (vehicle-treated mice [Ctrl] 2.69 ± 0.13 and elinogrel-treated mice [elinogrel] 1.75 ± 0.24; *P =* 0.0022; **Figure 2C**). In line with the EAE course in *P2ry12^-/-^* mice, the disease onset did not differ between the two groups (Ctrl 12.20 ± 0.114 and elinogrel 13.8 ± 0.12 days after immunization; *P =* 0.2221; **Figure 2D**) and the number of animals which developed the disease was only marginally affected as most mice in both groups developed clinical symptoms (**Figure 2E****).** Therefore, the phenotype of the elinogrel-treated mice mirrored the key clinical feature observed in the *P2ry12^-/-^* mice.

**Figure 2:**
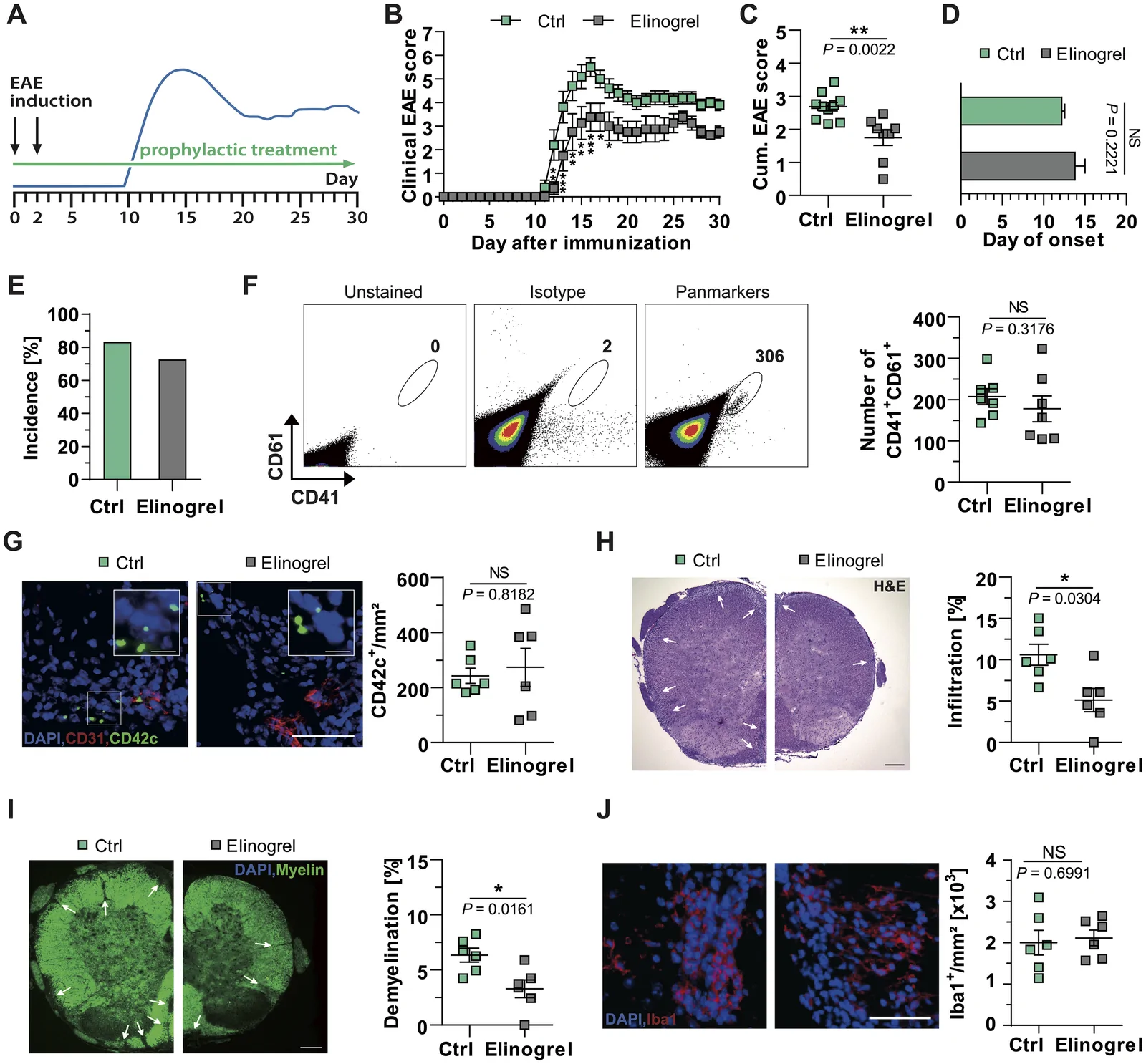
The impact of selective ADP receptor P2Y_12_ blockade during EAE. **(A)** Experimental design of active EAE induction with MOG_35–55_ peptide illustrating the time points of P2Y_12_ inhibitor administration (prophylactic treatment). **(B)** Clinical EAE scores of WT mice treated with elinogrel or vehicle as control (Ctrl) *via* oral gavage once daily beginning from the day of immunization are shown. **(C)** The total (Cum.) EAE score is presented for each group. **(D)** The mean day of disease onset is given for Ctrl and elinogrel-treated mice, defined as the first day with a score ≥ 1. **(E)** The percentage of animals that developed EAE symptoms following immunization in both groups is displayed. **(F)** Platelet-rich supernatants isolated from central nervous system (CNS) homogenates at the disease maximum of EAE (day 16) were stained for cluster of differentiation (CD)41 and CD61 or their corresponding isotype controls and examined by flow cytometry (n = 7 per experimental group). Representative dot plots are shown and total numbers of infiltrating CD41^+^CD61^+^ cells (identifying platelets) were quantified. **(G)** In addition, lumbar spinal cord sections were stained with CD31, CD42c, and DAPI to quantify platelet infiltration in CNS tissue at the EAE disease maximum using histopathology (n = 6 per experimental group). Immunofluorescent staining images (scale bar: 50 µm) and close-ups (scale bar: 10 µm) of Ctrl and elinogrel-treated groups are shown. **(H–J)** Lumbar spinal cord sections obtained at disease maximum (day 16) from Ctrl and elinogrel-treated mice were assessed histopathologically using H&E staining **(H)**, myelin staining **(I)** and Ionized calcium-binding adapter molecule 1 (Iba1) staining **(J)** (n = 6 per experimental group). DAPI was used for visualizing cell nuclei within immunofluorescent staining **(I, J)**. For graphical representation, one‐half of the spinal cord sections [scale bar, 200 µm; **(H, I)**] or representative areas of the spinal cord [scale bar, 50 µm; **(J)**] are shown from each group. Arrows indicate infiltrated **(H)** or demyelinated areas **(I)**. The corresponding quantifications are shown, respectively. All data were compiled from two **(B–E)** or three **(F, G, H, I, J)** independent experiments. Data were analyzed by a two-way ANOVA comparing EAE disease progression between control and elinogrel-treated mice over time followed by Bonferroni’s multiple-comparisons test at each individual time point (n = 8 [elinogrel] vs. 10 [control] mice; **(B)**) or two-tailed Mann-Whitney U-test **(C, D, F, G, H, I, J)**. Each symbol **(C, F, G, H, I, J)** represents an individual mouse. The level of significance was labeled as NS, *P < 0.05, **P < 0.01 or ***P < 0.001. ADP, adenosine diphosphate; ANOVA, analysis of variance; CD, cluster of differentiation; CNS, central nervous system; Ctrl, control; Cum, cumulative; EAE, experimental autoimmune encephalomyelitis; H&E, hematoxylin and eosin; Iba1, Ionized calcium-binding adapter molecule 1; MOG, myelin oligodendrocyte glycoprotein; NS, not significant; WT, wild-type.

Given prior evidence linking platelet paucity in the effector phase of EAE to a reduced EAE symptom burden (9, 11), the number of CNS-infiltrating platelets was assessed in elinogrel-treated and control mice. Flow cytometry analyses performed at the disease maximum showed no substantial difference between the two groups in the counts of CNS-infiltrating platelets (Ctrl 207.3 ± 19.5 and elinogrel 178.0 ± 31.5 CD41+CD61+ cells; *P* = 0.3176; **Figure 2F**). Consistently, immunohistological analyses of infiltrating platelets also confirmed no significant difference between the two groups (Ctrl 242.3 ± 27.71 and elinogrel 273.0 ± 68.90 CD42c^+^/mm^2^; *P =* 0.818; **Figure 2G**). Since immune cell infiltration and demyelination are hallmark features of the EAE pathology (6), immunohistochemical examinations of spinal cord sections obtained at the time of disease maximum were performed. Consistent with clinical scores, elinogrel-receiving mice demonstrated reduced cell infiltration, as assessed by H&E staining of lumbar spinal cord sections (Ctrl 10.61 ± 1.27% and elinogrel 5.13 ± 1.41%; *P =* 0.0304; **Figure 2H**). Accordingly, elinogrel-receiving mice showed a lower demyelinated lesion burden in the lumbar spinal cord (Ctrl 6.35 ± 0.63% and elinogrel 3.29 ± 0.81%; *P =* 0.0161; **Figure 2I**). Given that microglial activation is also a characteristic of EAE, and considering that P2Y_12_ is also expressed on microglia and implicated in their function (32), the impact of elinogrel treatment on these cells was evaluated. However, immunohistology did not detect significant differences in Iba1^+^ cell abundance between the two groups (Ctrl 2005 ± 299.1 and elinogrel 2118 ± 190.9 Iba1^+^/mm^2^; *P =* 0.6991; **Figure 2J**).

### 3.3 Elinogrel restrains the T_H_17 axis in EAE

As elinogrel treatment did not affect CNS platelet counts (**Figures 2F, G**), subsequent investigations focused on downstream immune correlates associated with disease modulation. Considering the pivotal involvement of pathogenic CD4^+^ T cell responses in MS and EAE (33), as well as documented interactions between platelets and CD4^+^ T cells during CNS autoimmunity (11), subsequent analyses focused on flow cytometric assessment of CNS T cell compartments and their activation states. Consistent with the histological findings, elinogrel-treated mice showed reduced numbers of CD45^high^ infiltrating leukocytes in the CNS at the disease peak (Ctrl 2227 ± 275.6 and elinogrel 1456 ± 431.5 CD45^high^ cells; *P =* 0.0379; **Figure 3A**). Flow cytometric characterization demonstrated a significant reduction in infiltrating CD4^+^ T cells in particular (Ctrl 756.7 ± 131.6 and elinogrel 278.1 ± 43.01 CD4^+^ T cells; *P =* 0.0023; **Figure 3B**). Further phenotyping of the CNS-infiltrating CD4^+^ T cell population revealed no significant differences in the proportion of anti-inflammatory regulatory T cells (Ctrl 26.79 ± 4.19% and elinogrel 28.32 ± 3.91% CD4^+^FoxP3^+^ T cells; *P =* 0.8857; **Figure 3C**) or pro-inflammatory T helper 1 (T_H_1) cells (IFN-γ^+^ IL-17A^-^ Ctrl 27.07 ± 8.537% and elinogrel 30.17 ± 7.9%; *P =* 0.6991; **Figure 3D**). However, a diminished T_H_17 immune response within the inflamed CNS of elinogrel-treated mice was observed (IFN-γ^-^ IL-17A^+^ Ctrl 7.05 ± 1.26% and elinogrel 2.79 ± 0.58%; *P =* 0.0260; **Figure 3D**). Consistent with the overall reduction in CNS-infiltrating CD4^+^ T cells, the absolute numbers of IFN-γ^+^IL-17A^-^ T_H_1 cells, IFN-γ^+^IL-17A^+^ cells, IFN-γ^-^IL-17A^+^ T_H_17 cells, and FoxP3^+^ regulatory T cells were lower in elinogrel-treated mice (**Supplementary Figure 2**). Thus, these findings demonstrate that elinogrel treatment reduced the absolute abundance of multiple CNS-infiltrating CD4^+^ T-cell subsets and was additionally associated with a selective reduction in the relative frequency of T_H_17 cells within the CD4^+^ T-cell population. In line with this, peripheral MOG recall assays of splenocytes isolated at peak disease showed selectively reduced IL-17A levels for cells from elinogrel-treated mice (**Supplementary Figure 3**).

**Figure 3:**
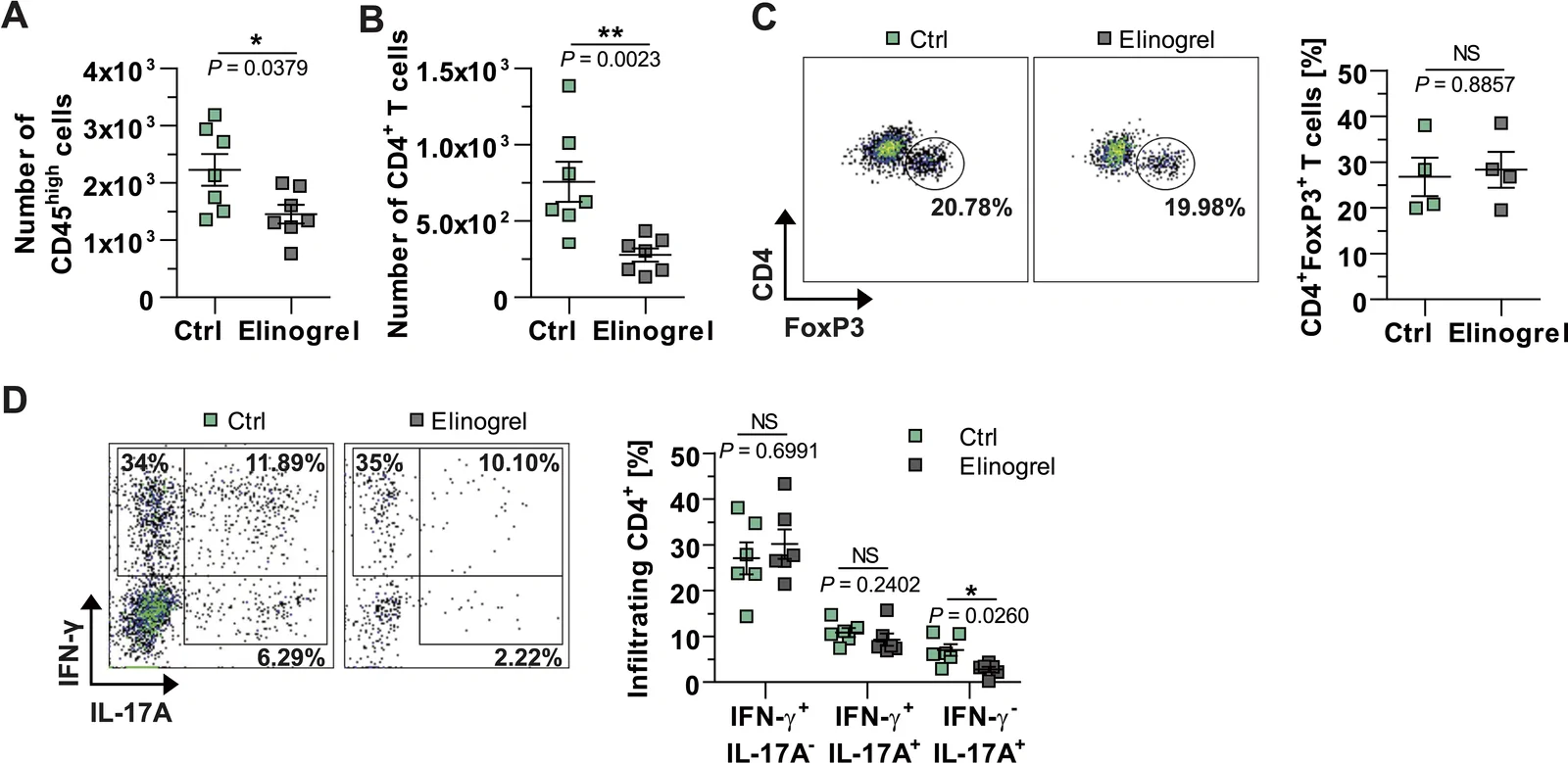
Targeted blockade of ADP receptor P2Y_12_ reduces CD4^+^ T cell infiltration into the CNS. **(A)** On day 16 of EAE, both Ctrl and elinogrel-treated mice were assessed for peripheral immune cell infiltration into the CNS using cell surface staining for CD45. The quantity of CD45^high^ cells was measured to determine the extent of infiltration. **(B)** Flow cytometry was used to measure CD4^+^ T cell counts at the peak of disease in each group, following staining for CD4 and CD3 markers. CNS-infiltrating CD4^+^ T cells were subjected to intranuclear staining for forkhead box protein P3 (FoxP3) (C) or intracellular staining for interferon-γ (IFN-γ) and interleukin-17A (IL-17A) **(D)**. The respective frequencies are expressed as a percentage of CD4^+^ T cells. Representative dot plots are shown **(C, D)**. All data were compiled from two **(C, D)** or three **(A, B)** independent experiments. Data were analyzed by two-tailed Mann-Whitney *U*-test. Each symbol represents an individual mouse [**(A, B)**: n = 7 per experimental group; **(C)**: n = 4 per group, **(D)**: n = 6 per group]. The level of significance was labeled as NS, *P < 0.05 or **P < 0.01. ADP, adenosine diphosphate; CD, cluster of differentiation; CNS, central nervous system; Ctrl, control; EAE, experimental autoimmune encephalomyelitis; FoxP3, Forkhead box protein P3; IFN, interferon; IL, interleukin; NS, not significant.

### 3.4 Pharmacological P2Y_12_ blockade ameliorates EAE progression prophylactically and therapeutically

To determine whether the observed beneficial effect of elinogrel is mediated through platelet P2Y_12_ signaling, an established platelet depletion regimen during the EAE effector phase (days 12 and 16) was combined with elinogrel, which was administered prophylactically starting on the day of immunization (day 0) (9). Elinogrel treatment in mice assigned to platelet depletion did not further improve the disease course compared with platelet depletion alone, indicating that platelet function contributes substantially to the clinical effect of elinogrel (cumulative EAE Score Ctrl 2.98 ± 0.11, platelet depletion 1.98 ± 0.16 and platelet depletion + elinogrel 2.07 ± 0.16; **Figure 4A**). Relative to control mice that received neither elinogrel nor platelet-depleting serum, both groups receiving platelet-depleting serum showed significantly reduced EAE severity (**Figure 4A**).

**Figure 4:**
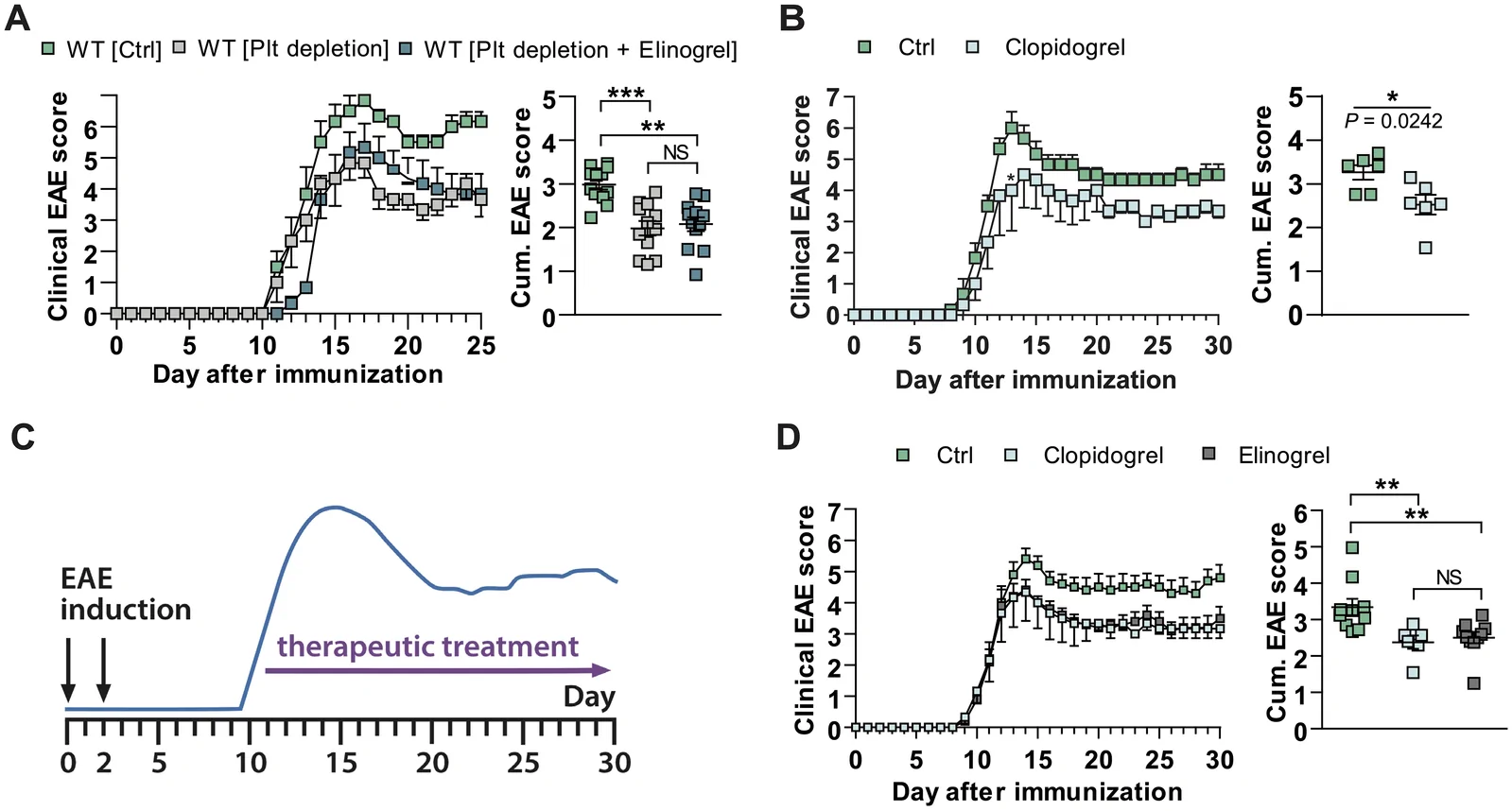
P2Y_12_ inhibition ameliorates EAE in prophylactic and therapeutic treatment regimens. **(A)** EAE was induced in age-matched WT mice. On days 12 and 16 after EAE induction, WT mice were injected with either the control serum (Ctrl) or the platelet-depleting serum (Plt depletion). In addition to anti-platelet serum administration, one group was treated with elinogrel *via* oral gavage once daily beginning on the day of immunization. **(B)** Clinical EAE scores of WT mice treated with clopidogrel or vehicle as control (Ctrl) *via* oral gavage once daily from the day of immunization are shown. **(C)** Schematic of the experimental design of active EAE induction with MOG_35–55_ peptide, showing P2Y_12_ inhibitor administration time for therapeutic treatment. **(D)** Clinical and cumulative (Cum.) EAE scores of WT mice treated with clopidogrel, elinogrel or vehicle as Ctrl *via* oral gavage once daily from the first day of clinical symptoms are displayed. **(A, B, D)** Scores for each day of all groups after immunization as well as their corresponding Cum. EAE scores are shown. All data were compiled from two independent experiments. Data were analyzed by two-way ANOVA comparing EAE disease progression between control and clopidogrel-treated mice over time followed by Bonferroni’s multiple-comparisons test at each individual time point (clinical scores: B left panel) or two-tailed Mann-Whitney U-test (cum. scores: B right panel), or a Kruskal–Wallis test followed by Dunn’s multiple-comparisons test for pairwise comparisons between groups (cum. scores: A, D right panel) (**(A)**: n = 12 mice per experimental group; **(B)**: n = 6 mice per experimental group; **(D)**: n = 6 [clopidogrel] vs. 10 [elinogrel] vs. 10 [control] mice per group). Each symbol represents an individual mouse (right panels in **A**, **B**, and **D**). The level of significance was labeled as NS, *P < 0.05, **P < 0.01, or *** *P* < 0.001. ANOVA, analysis of variance; Ctrl, control; Cum, cumulative; EAE, experimental autoimmune encephalomyelitis; MOG, myelin oligodendrocyte glycoprotein; NS, not significant; Plt, platelet; WT, wild-type.

To extend these findings toward clinical applicability, clopidogrel, an approved P2Y_12_ inhibitor, was evaluated for its ability to reproduce elinogrel-mediated protection in EAE (29). In a prophylactic treatment paradigm, clopidogrel similarly yielded an amelioration of neuroinflammatory processes and markedly attenuated disease severity (cumulative EAE Score Ctrl 3.27 ± 0.17 and clopidogrel 2.52 ± 0.22; *P =* 0.0242; **Figure 4B**). This finding is consistent with previous reports and supports the conclusion that the observed protection is a class-relevant effect of P2Y_12_ inhibition rather than an elinogrel-specific phenomenon.

To assess translational relevance beyond prophylactic dosing, treatment was initiated on the day of clinical onset according to a therapeutic regimen (**Figure 4C**). Under this post-onset scheme, both elinogrel and clopidogrel attenuated ongoing EAE severity (cumulative EAE Score Ctrl 3.35 ± 0.23, clopidogrel 2.38 ± 0.18 and elinogrel 2.51 ± 0.49; **Figure 4D**). Together, these data show that pharmacological P2Y_12_ blockade is effective not only prophylactically but also after neurological symptoms have already emerged.

## 4 Discussion

In this study, we investigated the contribution of platelet P2Y_12_ signaling to autoimmune neuroinflammation and demonstrated that both genetic ablation and pharmacological blockade confer protection in EAE. These findings support a role for P2Y_12_ signaling in neuroinflammation and are consistent with a substantial platelet-mediated contribution, although they do not establish this mechanism conclusively. Of note, the protective effect was not attributable to reduced disease incidence or delayed onset, but rather to a reduction in disease burden, as reflected by lower peak and cumulative disease severity. This pattern supports the concept that platelet P2Y_12_ signaling intensifies existing inflammatory tissue damage rather than being essential for disease priming (9, 25, 27). Mechanistically, this hypothesis seems plausible, as P2Y_12_ is a canonical ADP-sensitive receptor that both stabilizes and amplifies platelet activation (27). Additionally, ADP from activated platelets and injured vessels may drive local thrombo-inflammatory signaling at neurovascular sites (25).

The characterization of CNS infiltrates provided further insights into the immunological changes associated with P2Y_12_ blockade and the potential contribution of platelets to autoimmune neuroinflammation. Despite leaving CNS platelet counts unchanged, elinogrel markedly reduced CNS leukocyte infiltration, with fewer infiltrating CD4^+^ T cells and diminished T_H_17-related responses. One likely explanation is that P2Y_12_ inhibition predominantly modifies the platelet activation state and platelet-T cell interactions, rather than affecting platelet abundance within the CNS. Although the involvement of P2Y_12_ in EAE remains a subject of debate across various studies (24, 28), these results align with previous findings observed in EAE demonstrating that platelets have the ability to interact with CD4^+^ T cells and modulate their pathogenic functions during both the onset and progression of disease (11). Moreover, platelets are known to shape T cell cytokine profiles (17, 34). Accordingly, in peripheral MOG_35–55_ recall assays, IL-17A was suppressed without affecting other cytokines implicated in EAE pathology, suggesting preferential modulation of the T_H_17 axis. Collectively, these findings point to a potential role for platelet P2Y_12_ signaling in the qualitative modulation of immune responses, rather than merely decreasing platelet numbers within the CNS.

Our findings are consistent with earlier studies highlighting platelets as active inflammatory mediators in EAE and MS rather than passive bystanders (8–13) and showing that interventions targeting platelets are effective in reducing autoimmune CNS inflammation (9, 11). The protective effects observed in this study align with previous reports investigating P2Y_12_ antagonism in EAE. A study by Qin et al. reported that genetic P2Y_12_ deficiency reduced peak disease severity, leukocyte CNS infiltration, demyelination, and T_H_17 frequency, and that the clinically approved P2Y_12_ antagonists clopidogrel and ticagrelor alleviated EAE while suppressing pathogenic T_H_17 differentiation (24). These findings suggest that P2Y_12_ signalling can contribute to autoimmune pathology not only through platelet activation but also *via* direct modulation of adaptive immunity.

However, conflicting data exist, as another study found that global P2Y_12_ knockout aggravated EAE through increased IL-23 production by dendritic cells and enhanced T_H_17 differentiation (28). These observations emphasize that the role of P2Y_12_ is context-dependent and may differ across immune cell populations. Notably, in our study, IL-23 remained undetectable following MOG_35–55_-specific restimulation of splenocytes harvested from immunologically active peripheral organs at the peak of the disease in mice treated with elinogrel.

Prior EAE studies have found advantages in using P2Y_12_ antagonists such as clopidogrel and ticagrelor in dosing regimens that begin early or are used in a preventive manner (24). However, in this study, the therapeutic benefit of both elinogrel and clopidogrel was evident even when treatment was initiated after disease onset, suggesting that platelet P2Y_12_ signalling remains relevant beyond the earliest phases of EAE pathogenesis. Given the efficacy of pharmacological P2Y_12_ antagonism in our therapeutic intervention paradigm, which reflects clinical practice more closely, these results further underscore the translational potential of targeting P2Y_12_.

Importantly, the contribution of platelets to myelin repair may depend on the timing, duration, and localization of platelet exposure. Recent work in a focal toxin-induced lesion model showed that partial platelet depletion during the remyelination phase impaired OPC differentiation and subsequent remyelination. Transient platelet exposure promoted OPC differentiation *in vitro*, whereas sustained exposure or thrombocytosis was detrimental, suggesting that transient platelet recruitment may provide reparative signals, while persistent platelet accumulation may contribute to remyelination failure (35). Moreover, platelets were shown to enhance OPC differentiation and influence oligodendroglial populations primarily when in direct contact with these cells or present within the brain parenchyma, whereas thrombocytopenia without platelet infiltration had no comparable effect (36). Earlier findings that platelets can directly induce an OPC injury response provide additional evidence that oligodendroglial cells respond to platelet-derived signals (37). These findings caution against assuming that prolonged, stage-independent suppression of platelet function would remain beneficial throughout the disease course. Although the preservation of CNS platelet abundance despite reduced inflammatory pathology suggests that elinogrel-mediated P2Y_12_ blockade is biologically distinct from platelet depletion, it remains to be determined whether this intervention preserves or interferes with platelet-derived signals involved in oligodendroglial differentiation.

Beyond platelets, P2Y_12_ is also expressed on microglia, where it is considered a marker of homeostatic, surveillant phenotypes (32, 38). In both human MS lesions and EAE, microglial P2Y_12_ expression is downregulated during active inflammation and re-expressed during remission and recovery phases (39, 40). This dynamic regulation suggests that microglial P2Y_12_ may reflect reparative or anti-inflammatory states rather than driving pathogenic activation. Importantly, as elinogrel was developed as an antiplatelet P2Y_12_ inhibitor, its protective effects in this model are likely mediated predominantly by modulation of platelet-dependent inflammatory processes at the BBB rather than by direct effects on microglia (41). Moreover, the absence of an effect on immunohistological Iba1 staining further supports the hypothesis that the observed effects are mediated peripherally, potentially *via* platelets. Nevertheless, since BBB integrity is compromised during EAE, a direct effect of elinogrel on microglia cannot be completely excluded. This represents a limitation that should be addressed in future studies.

The absence of an additional benefit from elinogrel in mice receiving platelet-depleting serum is consistent with a predominantly platelet-dependent mechanism, but does not establish that both interventions act through an identical biological pathway. An important limitation of our study is that depletion efficiency and persistence were not verified in these cohorts by peripheral platelet counts or measurements of CNS-associated platelets after administration of an anti-platelet serum. The applied protocol was based on established antibody-mediated depletion paradigms. In EAE, we previously demonstrated that the same protocol reduced circulating platelet numbers by more than 97% within 24 hours, with depletion persisting for at least 72 hours, and markedly decreased the platelet-specific marker GPIb within the inflamed CNS (9). Consistent findings have been reported with antibody-mediated platelet depletion using anti-GPIbα/CD42b-based approaches, which produced marked reductions in circulating and CNS-associated platelets in EAE and other models of CNS pathology, including focal demyelination (13, 35, 42). Nevertheless, evidence from these established protocols cannot confirm effective and sustained depletion in the animals examined here. Incomplete or transient depletion, technical or biological variability, and a potential influence of concurrent elinogrel treatment on depletion efficiency therefore cannot be completely excluded, although the observed clinical effects are consistent with effective platelet depletion. Accordingly, the platelet-depletion experiment provides supportive but not definitive evidence for a predominantly platelet-dependent effect of elinogrel. A further limitation is that downstream phenotypes, including CNS T_H_17 responses and demyelination, were not directly compared across all treatment groups. In addition, the absence of a platelet-specific P2Y_12_ knockout model limits the attribution of the observed effects specifically to platelet P2Y_12_ signaling. Given that P2Y_12_ is expressed by multiple immune cell types (43, 44), systemic pharmacological inhibition does not allow attribution of the observed therapeutic effects solely to platelet signalling. Future studies employing cell-type-specific deletion strategies will be essential to dissect the relative contribution of platelet *versus* leukocyte P2Y_12_ signaling in autoimmune neuroinflammation. Furthermore, this study did not directly examine platelet-T cell interactions, as has been reported in other disease contexts, nor did it assess the integrity of the BBB, particularly given established associations between platelets and BBB endothelial cells (10, 17, 45). Future research should address these gaps to provide a more comprehensive understanding.

Overall, these data suggest that P2Y_12_ signaling contributes to the amplification of inflammation in MS-like diseases and that pharmacological inhibition of this pathway is associated with diminished CNS immune cell infiltration and attenuated T_H_17-associated responses. Although our findings are consistent with an important contribution of platelet P2Y_12_ signaling, the involvement of P2Y_12_ expressed by other cell types cannot be excluded. Since P2Y_12_ antagonists are already approved and widely used for vascular disorders such as myocardial infarction, repurposing these agents could offer a promising translational perspective for MS. However, further research is required to determine optimal timing, dosing, and long-term effects, particularly regarding recovery and remyelination phases. In addition, the dual role of P2Y_12_ in peripheral immunity and CNS homeostasis, together with the potential influence of sustained platelet inhibition on remyelination, highlights the need for careful therapeutic targeting and mechanistic understanding before clinical application (43, 44).

## Data Availability

The raw data supporting the conclusions of this article will be made available by the authors, without undue reservation.

## Supplementary material

Supplementary Figure 1 Elinogrel inhibits ADP-induced activation of platelets. **(A)** Murine platelets (n = 6) were left unstimulated (- ADP) or stimulated with ADP (+ ADP) in the presence of 0, 1, 10, or 100 µM elinogrel. Surface expression of CD62P and integrin αIIb was determined by flow cytometry and is reported as mean fluorescence intensity (MFI). Data are presented as mean ± SEM. ADP, adenosine diphosphate; CD, cluster of differentiation; MFI, mean fluorescence intensity.

Supplementary Figure 2 Absolute numbers of CNS-infiltrating CD4^+^ T-cell subsets under ADP receptor P2Y12 blockade. **(A)** On day 16 of EAE, CNS-infiltrating CD3⁺CD4⁺ T cells from control and elinogrel-treated mice were analyzed by flow cytometry. Absolute numbers of IFN-γ^+^IL-17A^-^, IFN-γ^+^IL-17A^+^, IFN-γ^-^IL-17A^+^, and FoxP3^+^CD4^+^ T cells are shown (n = 6 per group for IFN-γ^+^IL-17A^-^, IFN-γ⁺IL-17A^+^, IFN-γ^-^IL-17A^+^ subsets and n = 4 per group for FoxP3^+^ cells). Data are presented as mean ± SEM. CD, cluster of differentiation; CNS, central nervous system; Ctrl, control; EAE, experimental autoimmune encephalomyelitis; FoxP3, forkhead box protein P3; IFN, interferon; IL, interleukin; SEM, standard error of the mean.

Supplementary Figure 3 Elinogrel shapes peripheral antigen-specific immune responses in lymphoid tissues. **(A)** Active MOG_35–55_ EAE was induced in age-matched wild-type mice, which subsequently received prophylactic treatment once daily with either elinogrel or vehicle. At disease maximum, splenocytes were cultured for 48 h either unstimulated or restimulated with MOG_35–55_ (20 µg/mL). Proliferation was expressed as a proliferation index (stimulated/unstimulated). In parallel, splenocytes were restimulated for 48 h with MOG_35–55_ (10 µg/mL) and the following cytokines in supernatants were measured: Interleukin-6 (IL-6), Interleukin-10 (IL-10), Interleukin-17A (IL-17A), Interferon gamma (IFN-γ), Tumor necrosis factor alpha (TNF-α), Transforming growth factor beta 1 (TGF-β1), and Interleukin-23 (IL-23; detection limit: 15 pg/mL). All data were compiled from two independent experiments. Data were analyzed by two-tailed Mann-Whitney U-test. Each symbol represents an individual mouse. The level of significance was labeled as NS or *P < 0.05. EAE, experimental autoimmune encephalomyelitis; IFN, interferon; IL, interleukin; MOG, myelin oligodendrocyte glycoprotein; NS, not significant; Plt, platelet; TGF, transforming growth factor; TNF, tumor necrosis factor; WT, wild-type.

## Glossary

18S rRNA: 18S ribosomal RNA
ADP: Adenosine diphosphate
ANOVA: Analysis of variance
ARRIVE: Animal Research: Reporting of *In Vivo* Experiments
ATP: Adenosine triphosphate
BBB: Blood–brain barrier
BD: Becton, Dickinson and Company
BSA: Bovine serum albumin
CD: Cluster of differentiation
cDNA: complementary DNA
CMC: Carboxymethyl cellulose
CNS: Central nervous system
Ctrl: Control
Ct: Cycle threshold
Cum.: Cumulative
DAPI: 4′, 6-diamidino-2-phenylindole
DMSO: Dimethyl sulfoxide
DPBS: Dulbecco’s phosphate-buffered saline
EAE: Experimental autoimmune encephalomyelitis
ELISA: enzyme-linked immunosorbent assay
FCS: fetal calf serum
FoxP3: Forkhead box protein P3
H&E: Hematoxylin and eosin
Iba1: Ionized calcium-binding adapter molecule 1
IFN-γ: Interferon gamma
IL-6: Interleukin-6
IL-10: Interleukin-10
IL-17A: Interleukin-17A
IL-23: Interleukin-23
*i.p.*: Intraperitoneal
KO: Knockout
MBMEC: Murine brain microvascular endothelial cells
MFI: mean fluorescence intensity
MOG: Myelin oligodendrocyte glycoprotein
mRNA: Messenger RNA
MS: Multiple sclerosis
MΦ: Macrophages
NS: Not significant
O.C.T.: Optimal cutting temperature compound
OLG: Oligodendrocytes
OPC: Oligodendroglial precursor cells
*P2ry12*: *P2Y12* gene
P2Y_12_: Purinergic receptor P2Y12
PCR: Polymerase chain reaction
PFA: Paraformaldehyde
Plt: Platelets
PTx: Pertussis toxin
rRT-PCR: Real-time reverse transcription polymerase chain reaction
RT: Room temperature
SEM: Standard error of the mean
T_H_: T helper (cells)
T_H_1: T helper 1
T_H_17: T helper 17
TGF-β1: Transforming growth factor beta 1
TNF-α: Tumor necrosis factor alpha
WT: Wild-type
